# Theoretical Photoelectron Spectroscopy of Metal–Metal Quintuple Bonds: Relativity-Driven Reordering of Frontier Orbitals

**DOI:** 10.1021/acsorginorgau.4c00002

**Published:** 2024-03-01

**Authors:** Abhik Ghosh, Jeanet Conradie

**Affiliations:** † Department of Chemistry, UiT − The Arctic University of Norway, N-9037 Tromsø, Norway; ‡ Department of Chemistry, University of the Free State, P.O. Box 339, Bloemfontein 9300, South Africa

**Keywords:** quintuple bond, quadruple bond, photoelectron spectroscopy, density functional theory, amidinate

## Abstract

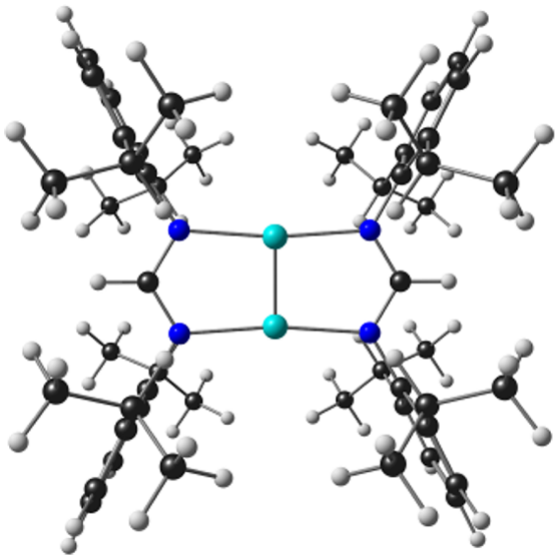

A recent reinvestigation of the gas-phase photoelectron spectra of Group 6 metal–metal quadruple-bonded complexes with scalar-relativistic DFT calculations showed that common exchange-correlation functionals reproduce the lowest ionization potentials in a semiquantitative manner. The finding encouraged us to undertake a DFT study of metal–metal quintuple bonds in a set of bisamidinato complexes with the formula M^I^_2_[HC(NR)_2_]_2_ (M = Cr, Mo, W; R = H, Ph, 2,6-*i*Pr_2_C_6_H_3_) and idealized *D*_2*h*_ symmetry. Scalar-relativistic OLYP/STO-TZ2P calculations indicated significant shifts in valence orbital energies among the three metals, which translate to lower first ionization potentials, higher electron affinities, and lower HOMO–LUMO gaps for the W complexes relative to their Cr and Mo counterparts. These differences are largely attributable to substantially larger relativistic effects in the case of tungsten relative to those of its lighter congeners.

Among the physical techniques used to study metal–metal quadruple bonds,^1^ gas-phase photoelectron spectroscopy (PES) was undoubtedly one of the most insightful. PES studies of Group 6 complexes with a variety of supporting ligands provided direct measures of the ionization potentials (IPs) of the σ, π, and δ bonds constituting the quadruple bonds.^2,3^ A fascinating finding to emerge from these studies is that the tungsten complex W_2_(Hpp)_4_ (Hpp = hexahydropyrimidinopyrimidine) is easier to ionize than atomic cesium!^4^ We recently revisited the PES data with scalar-relativistic density functional theory (DFT) calculations and found that common exchange-correlation functionals reproduce the lowest ionization potentials of such quadruple-bonded systems with semiquantitative accuracy.^5^ Together, the PES and DFT data provided a wealth of insights into periodic trends and relativistic effects,^6,7^ significantly deepening our appreciation of relativistic effects in coordination chemistry.^8−12^

During the first decade of the new millennium, Group 6 elements were also shown to sustain quintuple bonds in complexes such as RMMR, with R being a univalent group such as a sterically hindered aryl group.^13,14^ Curiously, the first such compound to be reported, ArCrCrAr (Ar being a sterically hindered terphenyl substituent), was found to exhibit a *trans*-bent geometry.^15^ Quantum chemical studies showed that the unexpected geometry corresponds to one of a number of local minima and that the deviation from a linear geometry does not significantly impact the integrity of the quintuple bond.^16−18^ Understandably, such structural ambiguities do not arise for bridged Group 6 complexes such as M_2_[HC(NR)_2_]_2_ (Scheme 1).^19−22^ The high, idealized *D*_2*h*_ local symmetry of the quintuple bonds in amidinate-bridged complexes allowed us to calculate four of their lowest IPs with conventional DFT^23−25^ calculations and simple group-theoretical manipulations (involving specification of the expected numbers of electrons under different irreducible representations). Throughout, we used a scalar-relativistic ZORA (Zeroth Order Regular Approximation to the Dirac equation)^26^ Hamiltonian, the well-tested OLYP^27,28^ exchange-correlation functional, augmented with D3^29,30^ dispersion corrections, all-electron ZORA-STO-TZ2P basis sets, fine integration grids, and tight criteria for SCF and geometry optimization cycles (which we carefully tested), as implemented in the ADF program system.^31^

**Scheme 1:**
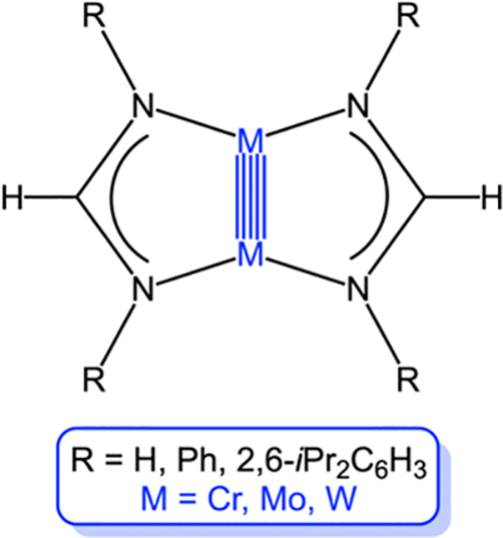
Amidinate-Bridged, Quintuple-Bonded Group 6 Metal(I) Complexes Studied in This Work

Figure 1 presents scalar-relativistic comparative energy level diagrams of three of the compounds studied, with M = Cr, Mo, W, and R = 2,6-*i*Pr_2_C_6_H_3_, with key molecular orbitals (MOs) visually depicted in Figure 2. Table 1 presents calculated ionization potentials (IP1–IP4 for R = 2,6-*i*Pr_2_C_6_H_3_), electron affinities (EAs), and singlet–triplet gaps for two different excited states of the quintuple bond, with all energies determined via a Δ*S*CF procedure, i.e., as differences in electronic energy between the two states of interest. In general, we found only small differences between vertical and adiabatic energies; the handful of cases where the energy difference exceeds 0.1 eV reflect small differences in ligand character in the open-shell orbital between the vertically and adiabatically ionized/excited states. The results lead to a fascinating set of predictions on periodic trends and relativistic effects, which may well justify an experimental PES study of the complexes.

**Figure 1:**
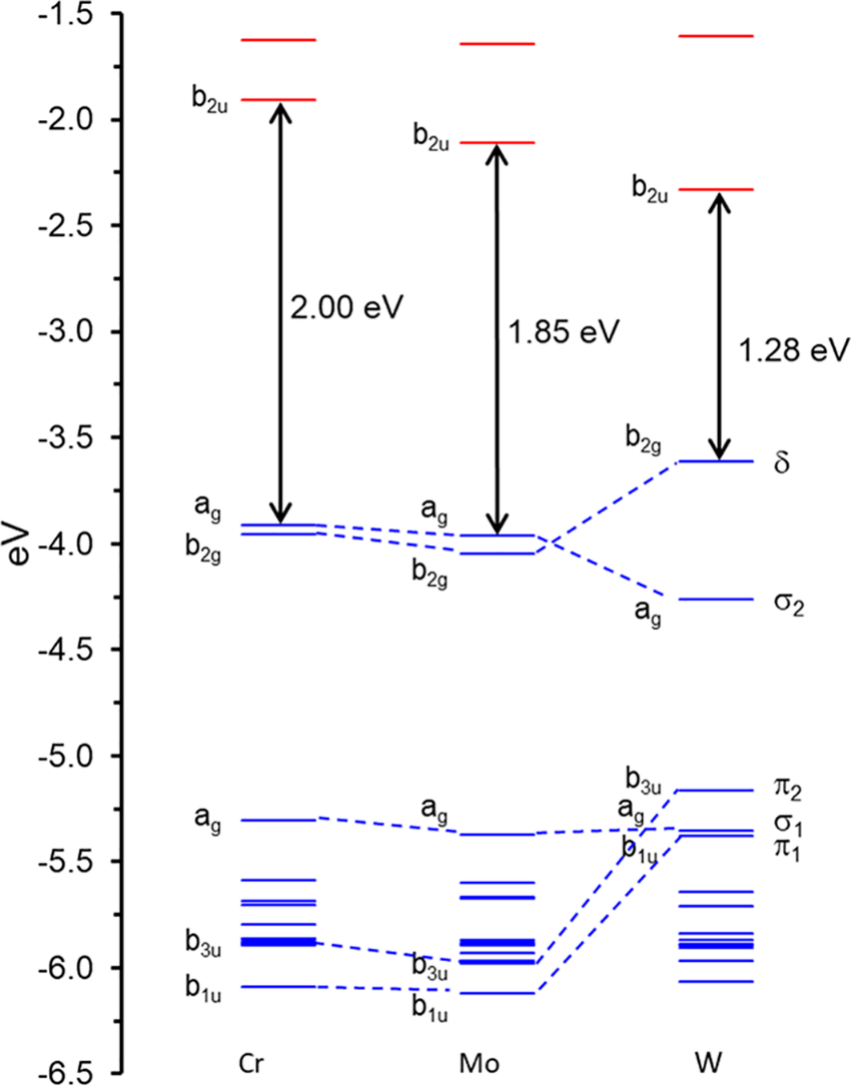
Comparative scalar-relativistic OLYP-D3/ZORA-STO-TZ2P MO energy level diagram for M_2_[HC(NR)_2_] (R = 2,6-*i*Pr_2_C_6_H_3_).

**Figure 2:**
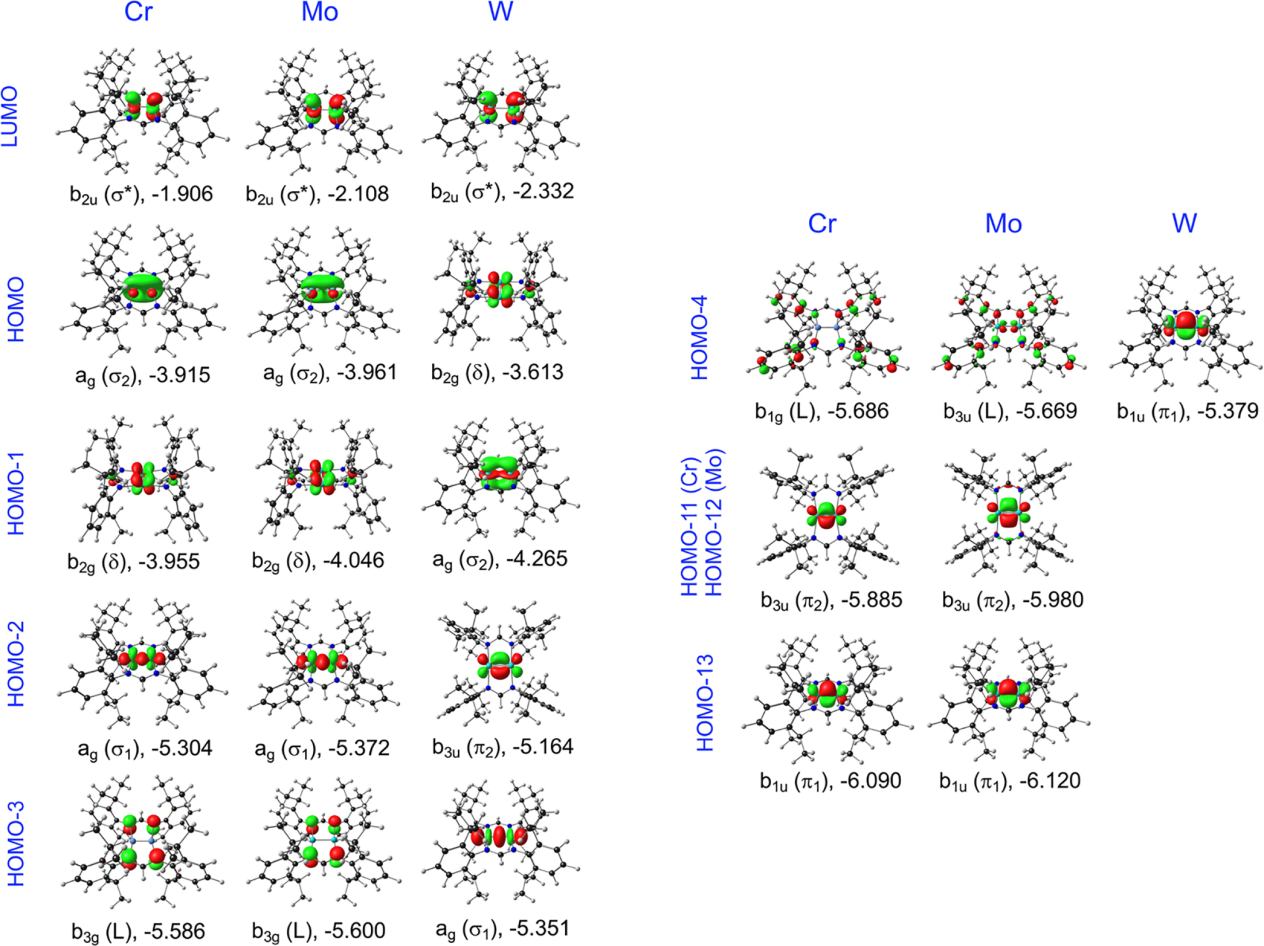
Visual depictions of key Kohn–Sham MOs included in Figure 1. The irrep, bonding character, and orbital energy (eV) of each MO are indicated; L = ligand.

**Table 1:** OLYP/ZORA-STO-TZ2P Ionization Potentials (IP1-IP4), Electron Affinities (EA), and Triplet Energies (T1 and T2) for M_2_ Complexes with the Experimentally Used HC(N-2,6-*i*Pr_2_C_6_H_3_)_2_ Ligand^a^

M	IP1	IP2	IP3	IP4	T1	T2	EA
Cr_2_[HC(N-2,6-*i*Pr_2_C_6_H_3_)_2_]_2_	*5.47*/5.41 (a_g_)	*5.67*/5.54 (b_2g_)	*6.78*/6.71 (b_1g_)	*6.84*/6.78 (b_3g_)	1.17 (σσ*)^a^	1.29 (δσ*)	*0.01*/0.09 (b_2u_)
Mo_2_[HC(N-2,6-*i*Pr_2_C_6_H_3_)_2_]_2_	*5.43*/5.40 (a_g_)	*5.67*/5.58 (b_2g_)	*6.78*/6.70 (b_3u_)	*6.86*/6.81 (b_3g_)	1.07 (σσ*)	1.37 (δσ*)^a^	*0.29*/0.39 (b_2u_)
W_2_[HC(N-2,6-*i*Pr_2_C_6_H_3_)_2_]_2_	*5.17*/5.11 (b_2g_)	*5.79*/5.71 (a_g_)	*6.56*/6.30 (b_3u_)	*6.84*/6.78 (b_1u_)	0.80 (δσ*)	0.86 (σσ*)	*0.58*/0.68 (b_2u_)

a All values are in eV and were obtained via a Δ*S*CF procedure. Vertical and adiabatic values are indicated in italics and normal script, respectively. The irreps refer to the *D*_2h_ point group and a given irrep refers to the MO from which an electron has been removed or to which an electron has been added.

The Cr and Mo complexes exhibit very similar energy levels (to within a couple of tenths of an eV) and HOMO–LUMO gaps. The MO energy levels of the W complex, in contrast, are significantly different (Figure 1). These differences are also reflected in the IPs, EAs, and triplet energies listed in Tables 1 and 2. Based on a large body of earlier studies,^8−12^ the majority of these difference may be ascribed to greater relativistic destabilization of the W(5d)-based energy levels relative to analogous Cr(3d)- and Mo(4d)-based energy levels. Indeed, switching off relativity in our calculations (while maintaining the same basis sets) resulted in very similar MO energy levels for all three metals.

**Table 2:** Selected OLYP/ZORA-STO-TZ2P Ionization Potentials, Electron Affinities (EA), and Triplet Energies (T1, σσ*) for the Molecules Studied with Simplified Amidinato Ligands^a^

R	M	IP1	T1	EA
Ph	Cr	*5.63*/5.63	1.18	0.09
	Mo	*5.56*/5.53	1.12	0.22
	W	*5.18*/5.13	0.78	0.46
H	Cr	*5.95*/5.96	1.17	–0.68
	Mo	*5.86*/5.85	1.09	–0.18
	W	*5.43*/5.40	0.65	0.08

a The comments in footnote *a* of Table 1 also apply here.

A notable twist is found for the highest occupied metal–metal σ-bonding MO, which is lower in energy for the W complex. Visually, the MO appears to originate from sideways overlap of two metal d_z2_ orbitals (the M–M vector being identified as the *x*-axis and the mean molecular plane as the *xy* plane). An examination of the atomic orbital composition of this MO, however, shows that it includes about 30–40% metal s character, consistent with the fully symmetric nature (a_g_) of the MO. Relativistic stabilization of the W(6s) orbital then provides a straightforward explanation of the low energy of this MO in the tungsten case.

A similar effect is also observed for the LUMOs, with the W complex exhibiting a lower energy LUMO (which translates to a higher electron affinity) relative to the corresponding Cr and Mo complexes. For all three metals, the LUMO, at first glance, appears to involve an antibonding d_z2_–d_z2_ combination but actually also involves substantial metal s character. In the case of tungsten, the LUMO has approximately 53% s character and relativistic stabilization of the W(6s) orbital wins out over relativistic destabilization of the W(4d) orbitals.

Thus, there is substantial reordering of quintuple bond orbitals between Mo and W (as depicted in Figure 1), which translates to significant variations in the calculated valence ionization potentials among the compounds (Tables 1 and 2). Relativistic stabilization and destabilization of key orbitals also explain why the W complexes should exhibit both the highest electron affinity and the lowest singlet–triplet gaps for the three metals considered.

A technical point worth addressing is the accuracy of the data presented in Tables 1 and 2. In our laboratory, we have long known that DFT-based ΔSCF calculations do an excellent job of reproducing the lower IPs of organic compounds;^32−35^ there is less information available, however, for transition metal complexes.^36,37^ A comparison of calculated IPs with gas-phase PES data for Group 6 quadruple-bonded complexes suggests that the present values are likely to be slight underestimates relative to experimental values, by a margin of a few tenths of an eV (<0.5 eV).^5^ On the other hand, *differences* in calculated IPs among the different compounds studied should be almost quantitatively accurate, i.e., agree to within ∼0.1 eV with experimental values.^38,39^ We have less experience with DFT calculations of EAs,^40−42^ but given the large basis sets employed here, we may *a priori* expect a similar level of accuracy for EAs as well.

A final observation concerns the influence of the *N*-aryl groups on the formamidinate nitrogens. Without the aryl groups, the first adiabatic IPs are about a half an eV higher, while the EAs are about half an eV lower (see Tables 1 and 2). Such ligand substituent effects are expected and have been documented for Group 6 quadruple-bonded complexes^2−5^ as well as, in our own laboratory, for porphyrins and related macrocycles.^38,39^

In summary, scalar-relativistic DFT calculations predict substantial differences in the valence energy levels of quintuple-bonded Group 6 metal complexes, with significant relativity-driven orbital reordering between Mo and W. We remain intrigued by the possibility of experimental verification of the above results by means of gas-phase photoelectron spectroscopy.

## Data Availability

The data underlying this study are available in the published article and its Supporting Information.

## Supporting Information Available

Optimized DFT coordinates and sample input file (PDF)
