# Supplementary material for: Theoretical Photoelectron Spectroscopy of Metal–Metal Quintuple Bonds: Relativity-Driven Reordering of Frontier Orbitals
Source: ACS Org Inorg Au. 2024 Mar 1;4(3):301–5. doi: 10.1021/acsorginorgau.4c00002 (PMC11157506; doi:10.1021/acsorginorgau.4c00002)
Supplement: Supplementary file 1 — gg4c00002_si_001.pdf [file gg4c00002_si_001.pdf]

Supporting information

Theoretical Photoelectron Spectroscopy of Metal-Metal Quintuple Bonds: Relativity-Driven Reordering of Frontier Orbitals

Abhik Ghosh[a,*] and Jeanet Conradie[a,b,*]

[a] Department of Chemistry, UiT – The Arctic University of Norway, N-9037 Tromsø, Norway; Email: abhik.ghosh@uit.no

[b] Department of Chemistry, University of the Free State, P.O. Box 339, Bloemfontein, 9300, South Africa; Email: conradj@ufs.ac.za

OLYP/ZORA-STO-TZ2P optimized Cartesian coordinates (Å)

1. $Cr_2[HC\{N(iPr_2C_6H_3)\}_2]_2$, D_{2h}, q = 0, S = 0 2
2. $Mo_2[HC\{N(iPr_2C_6H_3)\}_2]_2$, D_{2h}, q = 0, S = 0 4
3. $W_2[HC\{N(iPr_2C_6H_3)\}_2]_2$, D_{2h}, q = 0, S = 0 7
4. $Cr_2[HC(NPh)_2]_2$, D_{2h}, q = 0, S = 0 9
5. $Mo_2[HC(NPh)_2]_2$, D_{2h}, q = 0, S = 0 10
6. $W_2[HC(NPh)_2]_2$, D_{2h}, q = 0, S = 0 11
7. $Cr_2[HC(NH)_2]_2$, D_{2h}, q = 0, S = 0 12
8. $Mo_2[HC(NH)_2]_2$, D_{2h}, q = 0, S = 0 13
9. $W_2[HC(NH)_2]_2$, D_{2h}, q = 0, S = 0 13
10. Example input for molecule with q = 0, S = 0.................... 14

1. $Cr_2[HC\{N(iPr_2C_6H_3)\}_2]_2$, D_{2h}, q = 0, S = 0

Cr	0.000000000	-0.827504000	0.000000000
Cr	0.000000000	0.827504000	0.000000000
C	1.968660000	3.228284000	3.586174000
C	1.968660000	3.228284000	-3.586174000
C	1.968660000	-3.228284000	3.586174000
C	1.968660000	-3.228284000	-3.586174000
C	2.618645000	2.404217000	0.000000000
C	2.618645000	-2.404217000	0.000000000
C	2.664755000	2.338785000	2.548655000
C	2.664755000	2.338785000	-2.548655000
C	2.664755000	-2.338785000	2.548655000
C	2.664755000	-2.338785000	-2.548655000
C	2.675690000	0.000000000	0.000000000
C	2.895659000	3.044439000	1.227172000
C	2.895659000	3.044439000	-1.227172000
C	2.895659000	-3.044439000	1.227172000
C	2.895659000	-3.044439000	-1.227172000
C	3.444293000	4.329772000	1.203502000
C	3.444293000	4.329772000	-1.203502000
C	3.444293000	-4.329772000	1.203502000
C	3.444293000	-4.329772000	-1.203502000
C	3.715657000	4.974586000	0.000000000
C	3.715657000	-4.974586000	0.000000000
C	4.001570000	1.820454000	3.102589000
C	4.001570000	1.820454000	-3.102589000
C	4.001570000	-1.820454000	3.102589000
C	4.001570000	-1.820454000	-3.102589000
C	-1.968660000	3.228284000	3.586174000
C	-1.968660000	3.228284000	-3.586174000
C	-1.968660000	-3.228284000	3.586174000
C	-1.968660000	-3.228284000	-3.586174000
C	-2.618645000	2.404217000	0.000000000
C	-2.618645000	-2.404217000	0.000000000
C	-2.664755000	2.338785000	2.548655000
C	-2.664755000	2.338785000	-2.548655000
C	-2.664755000	-2.338785000	2.548655000
C	-2.664755000	-2.338785000	-2.548655000
C	-2.675690000	0.000000000	0.000000000
C	-2.895659000	3.044439000	1.227172000
C	-2.895659000	3.044439000	-1.227172000
C	-2.895659000	-3.044439000	1.227172000
C	-2.895659000	-3.044439000	-1.227172000
C	-3.444293000	4.329772000	1.203502000
C	-3.444293000	4.329772000	-1.203502000
C	-3.444293000	-4.329772000	1.203502000
C	-3.444293000	-4.329772000	-1.203502000
C	-3.715657000	4.974586000	0.000000000
C	-3.715657000	-4.974586000	0.000000000
C	-4.001570000	1.820454000	3.102589000
C	-4.001570000	1.820454000	-3.102589000

C	-4.001570000	-1.820454000	3.102589000
C	-4.001570000	-1.820454000	-3.102589000
H	1.075603000	3.694003000	3.165440000
H	1.075603000	3.694003000	-3.165440000
H	1.075603000	-3.694003000	3.165440000
H	1.075603000	-3.694003000	-3.165440000
H	1.675994000	2.631193000	4.457055000
H	1.675994000	2.631193000	-4.457055000
H	1.675994000	-2.631193000	4.457055000
H	1.675994000	-2.631193000	-4.457055000
H	2.020232000	1.476263000	2.354348000
H	2.020232000	1.476263000	-2.354348000
H	2.020232000	-1.476263000	2.354348000
H	2.020232000	-1.476263000	-2.354348000
H	2.634666000	4.023769000	3.935877000
H	2.634666000	4.023769000	-3.935877000
H	2.634666000	-4.023769000	3.935877000
H	2.634666000	-4.023769000	-3.935877000
H	3.668256000	4.829285000	2.141188000
H	3.668256000	4.829285000	-2.141188000
H	3.668256000	-4.829285000	2.141188000
H	3.668256000	-4.829285000	-2.141188000
H	3.768167000	0.000000000	0.000000000
H	3.843934000	1.265277000	4.032971000
H	3.843934000	1.265277000	-4.032971000
H	3.843934000	-1.265277000	4.032971000
H	3.843934000	-1.265277000	-4.032971000
H	4.141168000	5.975760000	0.000000000
H	4.141168000	-5.975760000	0.000000000
H	4.494815000	1.162998000	2.383341000
H	4.494815000	1.162998000	-2.383341000
H	4.494815000	-1.162998000	2.383341000
H	4.494815000	-1.162998000	-2.383341000
H	4.669773000	2.664101000	3.307925000
H	4.669773000	2.664101000	-3.307925000
H	4.669773000	-2.664101000	3.307925000
H	4.669773000	-2.664101000	-3.307925000
H	-1.075603000	3.694003000	3.165440000
H	-1.075603000	3.694003000	-3.165440000
H	-1.075603000	-3.694003000	3.165440000
H	-1.075603000	-3.694003000	-3.165440000
H	-1.675994000	2.631193000	4.457055000
H	-1.675994000	2.631193000	-4.457055000
H	-1.675994000	-2.631193000	4.457055000
H	-1.675994000	-2.631193000	-4.457055000
H	-2.020232000	1.476263000	2.354348000
H	-2.020232000	1.476263000	-2.354348000
H	-2.020232000	-1.476263000	2.354348000
H	-2.020232000	-1.476263000	-2.354348000
H	-2.634666000	4.023769000	3.935877000
H	-2.634666000	4.023769000	-3.935877000

H	-2.634666000	-4.023769000	3.935877000
H	-2.634666000	-4.023769000	-3.935877000
H	-3.668256000	4.829285000	2.141188000
H	-3.668256000	4.829285000	-2.141188000
H	-3.668256000	-4.829285000	2.141188000
H	-3.668256000	-4.829285000	-2.141188000
H	-3.768167000	0.000000000	0.000000000
H	-3.843934000	1.265277000	4.032971000
H	-3.843934000	1.265277000	-4.032971000
H	-3.843934000	-1.265277000	4.032971000
H	-3.843934000	-1.265277000	-4.032971000
H	-4.141168000	5.975760000	0.000000000
H	-4.141168000	-5.975760000	0.000000000
H	-4.494815000	1.162998000	2.383341000
H	-4.494815000	1.162998000	-2.383341000
H	-4.494815000	-1.162998000	2.383341000
H	-4.494815000	-1.162998000	-2.383341000
H	-4.669773000	2.664101000	3.307925000
H	-4.669773000	2.664101000	-3.307925000
H	-4.669773000	-2.664101000	3.307925000
H	-4.669773000	-2.664101000	-3.307925000
N	1.977294000	1.132158000	0.000000000
N	1.977294000	-1.132158000	0.000000000
N	-1.977294000	1.132158000	0.000000000
N	-1.977294000	-1.132158000	0.000000000

2. $Mo_2[HC\{N(iPr_2C_6H_3)\}_2]_2$, D_{2h}, q = 0, *S* = 0

Mo	0.000000000	-0.997400000	0.000000000
Mo	0.000000000	0.997400000	0.000000000
C	1.999666000	3.235385000	3.473113000
C	1.999666000	3.235385000	-3.473113000
C	1.999666000	-3.235385000	3.473113000
C	1.999666000	-3.235385000	-3.473113000
C	2.803330000	0.000000000	0.000000000
C	2.826046000	2.384636000	0.000000000
C	2.826046000	-2.384636000	0.000000000
C	2.832172000	2.332128000	2.552822000
C	2.832172000	2.332128000	-2.552822000
C	2.832172000	-2.332128000	2.552822000
C	2.832172000	-2.332128000	-2.552822000
C	3.141874000	3.003630000	1.228106000
C	3.141874000	3.003630000	-1.228106000
C	3.141874000	-3.003630000	1.228106000
C	3.141874000	-3.003630000	-1.228106000
C	3.774378000	4.250216000	1.203922000
C	3.774378000	4.250216000	-1.203922000
C	3.774378000	-4.250216000	1.203922000
C	3.774378000	-4.250216000	-1.203922000
C	4.089095000	4.874876000	0.000000000
C	4.089095000	-4.874876000	0.000000000

C	4.126071000	1.887249000	3.252158000
C	4.126071000	1.887249000	-3.252158000
C	4.126071000	-1.887249000	3.252158000
C	4.126071000	-1.887249000	-3.252158000
C	-1.999666000	3.235385000	3.473113000
C	-1.999666000	3.235385000	-3.473113000
C	-1.999666000	-3.235385000	3.473113000
C	-1.999666000	-3.235385000	-3.473113000
C	-2.803330000	0.000000000	0.000000000
C	-2.826046000	2.384636000	0.000000000
C	-2.826046000	-2.384636000	0.000000000
C	-2.832172000	2.332128000	2.552822000
C	-2.832172000	2.332128000	-2.552822000
C	-2.832172000	-2.332128000	2.552822000
C	-2.832172000	-2.332128000	-2.552822000
C	-3.141874000	3.003630000	1.228106000
C	-3.141874000	3.003630000	-1.228106000
C	-3.141874000	-3.003630000	1.228106000
C	-3.141874000	-3.003630000	-1.228106000
C	-3.774378000	4.250216000	1.203922000
C	-3.774378000	4.250216000	-1.203922000
C	-3.774378000	-4.250216000	1.203922000
C	-3.774378000	-4.250216000	-1.203922000
C	-4.089095000	4.874876000	0.000000000
C	-4.089095000	-4.874876000	0.000000000
C	-4.126071000	1.887249000	3.252158000
C	-4.126071000	1.887249000	-3.252158000
C	-4.126071000	-1.887249000	3.252158000
C	-4.126071000	-1.887249000	-3.252158000
H	1.103200000	3.590637000	2.959926000
H	1.103200000	3.590637000	-2.959926000
H	1.103200000	-3.590637000	2.959926000
H	1.103200000	-3.590637000	-2.959926000
H	1.693135000	2.683163000	4.368728000
H	1.693135000	2.683163000	-4.368728000
H	1.693135000	-2.683163000	4.368728000
H	1.693135000	-2.683163000	-4.368728000
H	2.239830000	1.438285000	2.340254000
H	2.239830000	1.438285000	-2.340254000
H	2.239830000	-1.438285000	2.340254000
H	2.239830000	-1.438285000	-2.340254000
H	2.576016000	4.109117000	3.794425000
H	2.576016000	4.109117000	-3.794425000
H	2.576016000	-4.109117000	3.794425000
H	2.576016000	-4.109117000	-3.794425000
H	3.894963000	1.353838000	4.180580000
H	3.894963000	1.353838000	-4.180580000
H	3.894963000	-1.353838000	4.180580000
H	3.894963000	-1.353838000	-4.180580000
H	3.895706000	0.000000000	0.000000000
H	4.028502000	4.734756000	2.142407000

H	4.028502000	4.734756000	-2.142407000
H	4.028502000	-4.734756000	2.142407000
H	4.028502000	-4.734756000	-2.142407000
H	4.582364000	5.844445000	0.000000000
H	4.582364000	-5.844445000	0.000000000
H	4.710433000	1.225206000	2.607666000
H	4.710433000	1.225206000	-2.607666000
H	4.710433000	-1.225206000	2.607666000
H	4.710433000	-1.225206000	-2.607666000
H	4.744048000	2.757880000	3.497349000
H	4.744048000	2.757880000	-3.497349000
H	4.744048000	-2.757880000	3.497349000
H	4.744048000	-2.757880000	-3.497349000
H	-1.103200000	3.590637000	2.959926000
H	-1.103200000	3.590637000	-2.959926000
H	-1.103200000	-3.590637000	2.959926000
H	-1.103200000	-3.590637000	-2.959926000
H	-1.693135000	2.683163000	4.368728000
H	-1.693135000	2.683163000	-4.368728000
H	-1.693135000	-2.683163000	4.368728000
H	-1.693135000	-2.683163000	-4.368728000
H	-2.239830000	1.438285000	2.340254000
H	-2.239830000	1.438285000	-2.340254000
H	-2.239830000	-1.438285000	2.340254000
H	-2.239830000	-1.438285000	-2.340254000
H	-2.576016000	4.109117000	3.794425000
H	-2.576016000	4.109117000	-3.794425000
H	-2.576016000	-4.109117000	3.794425000
H	-2.576016000	-4.109117000	-3.794425000
H	-3.894963000	1.353838000	4.180580000
H	-3.894963000	1.353838000	-4.180580000
H	-3.894963000	-1.353838000	4.180580000
H	-3.894963000	-1.353838000	-4.180580000
H	-3.895706000	0.000000000	0.000000000
H	-4.028502000	4.734756000	2.142407000
H	-4.028502000	4.734756000	-2.142407000
H	-4.028502000	-4.734756000	2.142407000
H	-4.028502000	-4.734756000	-2.142407000
H	-4.582364000	5.844445000	0.000000000
H	-4.582364000	-5.844445000	0.000000000
H	-4.710433000	1.225206000	2.607666000
H	-4.710433000	1.225206000	-2.607666000
H	-4.710433000	-1.225206000	2.607666000
H	-4.710433000	-1.225206000	-2.607666000
H	-4.744048000	2.757880000	3.497349000
H	-4.744048000	2.757880000	-3.497349000
H	-4.744048000	-2.757880000	3.497349000
H	-4.744048000	-2.757880000	-3.497349000
N	2.119042000	1.143356000	0.000000000
N	2.119042000	-1.143356000	0.000000000
N	-2.119042000	1.143356000	0.000000000

N	-2.119042000	-1.143356000	0.000000000

3. $W_2[HC\{N(iPr_2C_6H_3)\}_2]_2$, D_{2h}, q = 0, *S* = 0

W	0.000000000	-1.049859000	0.000000000
W	0.000000000	1.049859000	0.000000000
C	1.960571000	3.249603000	3.442777000
C	1.960571000	3.249603000	-3.442777000
C	1.960571000	-3.249603000	3.442777000
C	1.960571000	-3.249603000	-3.442777000
C	2.777743000	0.000000000	0.000000000
C	2.797740000	2.326179000	2.547917000
C	2.797740000	2.326179000	-2.547917000
C	2.797740000	-2.326179000	2.547917000
C	2.797740000	-2.326179000	-2.547917000
C	2.815203000	2.378349000	0.000000000
C	2.815203000	-2.378349000	0.000000000
C	3.142924000	2.987054000	1.227763000
C	3.142924000	2.987054000	-1.227763000
C	3.142924000	-2.987054000	1.227763000
C	3.142924000	-2.987054000	-1.227763000
C	3.814905000	4.213354000	1.204676000
C	3.814905000	4.213354000	-1.204676000
C	3.814905000	-4.213354000	1.204676000
C	3.814905000	-4.213354000	-1.204676000
C	4.069092000	1.866969000	3.277267000
C	4.069092000	1.866969000	-3.277267000
C	4.069092000	-1.866969000	3.277267000
C	4.069092000	-1.866969000	-3.277267000
C	4.151638000	4.825619000	0.000000000
C	4.151638000	-4.825619000	0.000000000
C	-1.960571000	3.249603000	3.442777000
C	-1.960571000	3.249603000	-3.442777000
C	-1.960571000	-3.249603000	3.442777000
C	-1.960571000	-3.249603000	-3.442777000
C	-2.777743000	0.000000000	0.000000000
C	-2.797740000	2.326179000	2.547917000
C	-2.797740000	2.326179000	-2.547917000
C	-2.797740000	-2.326179000	2.547917000
C	-2.797740000	-2.326179000	-2.547917000
C	-2.815203000	2.378349000	0.000000000
C	-2.815203000	-2.378349000	0.000000000
C	-3.142924000	2.987054000	1.227763000
C	-3.142924000	2.987054000	-1.227763000
C	-3.142924000	-2.987054000	1.227763000
C	-3.142924000	-2.987054000	-1.227763000
C	-3.814905000	4.213354000	1.204676000
C	-3.814905000	4.213354000	-1.204676000
C	-3.814905000	-4.213354000	1.204676000
C	-3.814905000	-4.213354000	-1.204676000
C	-4.069092000	1.866969000	3.277267000

C	-4.069092000	1.866969000	-3.277267000
C	-4.069092000	-1.866969000	3.277267000
C	-4.069092000	-1.866969000	-3.277267000
C	-4.151638000	4.825619000	0.000000000
C	-4.151638000	-4.825619000	0.000000000
H	1.082198000	3.614332000	2.906206000
H	1.082198000	3.614332000	-2.906206000
H	1.082198000	-3.614332000	2.906206000
H	1.082198000	-3.614332000	-2.906206000
H	1.624970000	2.710376000	4.335739000
H	1.624970000	2.710376000	-4.335739000
H	1.624970000	-2.710376000	4.335739000
H	1.624970000	-2.710376000	-4.335739000
H	2.194177000	1.441905000	2.324486000
H	2.194177000	1.441905000	-2.324486000
H	2.194177000	-1.441905000	2.324486000
H	2.194177000	-1.441905000	-2.324486000
H	2.543718000	4.117414000	3.768165000
H	2.543718000	4.117414000	-3.768165000
H	2.543718000	-4.117414000	3.768165000
H	2.543718000	-4.117414000	-3.768165000
H	3.809858000	1.334513000	4.198584000
H	3.809858000	1.334513000	-4.198584000
H	3.809858000	-1.334513000	4.198584000
H	3.809858000	-1.334513000	-4.198584000
H	3.867071000	0.000000000	0.000000000
H	4.080455000	4.691761000	2.143190000
H	4.080455000	4.691761000	-2.143190000
H	4.080455000	-4.691761000	2.143190000
H	4.080455000	-4.691761000	-2.143190000
H	4.661783000	1.200059000	2.646037000
H	4.661783000	1.200059000	-2.646037000
H	4.661783000	-1.200059000	2.646037000
H	4.661783000	-1.200059000	-2.646037000
H	4.675280000	5.779267000	0.000000000
H	4.675280000	-5.779267000	0.000000000
H	4.690278000	2.730373000	3.539523000
H	4.690278000	2.730373000	-3.539523000
H	4.690278000	-2.730373000	3.539523000
H	4.690278000	-2.730373000	-3.539523000
H	-1.082198000	3.614332000	2.906206000
H	-1.082198000	3.614332000	-2.906206000
H	-1.082198000	-3.614332000	2.906206000
H	-1.082198000	-3.614332000	-2.906206000
H	-1.624970000	2.710376000	4.335739000
H	-1.624970000	2.710376000	-4.335739000
H	-1.624970000	-2.710376000	4.335739000
H	-1.624970000	-2.710376000	-4.335739000
H	-2.194177000	1.441905000	2.324486000
H	-2.194177000	1.441905000	-2.324486000
H	-2.194177000	-1.441905000	2.324486000

H	-2.194177000	-1.441905000	-2.324486000
H	-2.543718000	4.117414000	3.768165000
H	-2.543718000	4.117414000	-3.768165000
H	-2.543718000	-4.117414000	3.768165000
H	-2.543718000	-4.117414000	-3.768165000
H	-3.809858000	1.334513000	4.198584000
H	-3.809858000	1.334513000	-4.198584000
H	-3.809858000	-1.334513000	4.198584000
H	-3.809858000	-1.334513000	-4.198584000
H	-3.867071000	0.000000000	0.000000000
H	-4.080455000	4.691761000	2.143190000
H	-4.080455000	4.691761000	-2.143190000
H	-4.080455000	-4.691761000	2.143190000
H	-4.080455000	-4.691761000	-2.143190000
H	-4.661783000	1.200059000	2.646037000
H	-4.661783000	1.200059000	-2.646037000
H	-4.661783000	-1.200059000	2.646037000
H	-4.661783000	-1.200059000	-2.646037000
H	-4.675280000	5.779267000	0.000000000
H	-4.675280000	-5.779267000	0.000000000
H	-4.690278000	2.730373000	3.539523000
H	-4.690278000	2.730373000	-3.539523000
H	-4.690278000	-2.730373000	3.539523000
H	-4.690278000	-2.730373000	-3.539523000
N	2.085092000	1.146482000	0.000000000
N	2.085092000	-1.146482000	0.000000000
N	-2.085092000	1.146482000	0.000000000
N	-2.085092000	-1.146482000	0.000000000

4. $Cr_2[HC(NPh)_2]_2$, D_{2h}, q = 0, S = 0

Cr	0.000000000	0.000000000	0.826963000
Cr	0.000000000	0.000000000	-0.826963000
C	0.000000000	2.669369000	0.000000000
C	0.000000000	2.676615000	2.369879000
C	0.000000000	2.676615000	-2.369879000
C	0.000000000	3.999908000	4.841892000
C	0.000000000	3.999908000	-4.841892000
C	0.000000000	-2.669369000	0.000000000
C	0.000000000	-2.676615000	2.369879000
C	0.000000000	-2.676615000	-2.369879000
C	0.000000000	-3.999908000	4.841892000
C	0.000000000	-3.999908000	-4.841892000
C	1.207705000	3.668607000	4.222510000
C	1.207705000	3.668607000	-4.222510000
C	1.207705000	-3.668607000	4.222510000
C	1.207705000	-3.668607000	-4.222510000
C	1.209792000	3.008733000	2.993086000
C	1.209792000	3.008733000	-2.993086000
C	1.209792000	-3.008733000	2.993086000
C	1.209792000	-3.008733000	-2.993086000

C	-1.207705000	3.668607000	4.222510000
C	-1.207705000	3.668607000	-4.222510000
C	-1.207705000	-3.668607000	4.222510000
C	-1.207705000	-3.668607000	-4.222510000
C	-1.209792000	3.008733000	2.993086000
C	-1.209792000	3.008733000	-2.993086000
C	-1.209792000	-3.008733000	2.993086000
C	-1.209792000	-3.008733000	-2.993086000
H	0.000000000	3.763351000	0.000000000
H	0.000000000	4.509759000	5.802374000
H	0.000000000	4.509759000	-5.802374000
H	0.000000000	-3.763351000	0.000000000
H	0.000000000	-4.509759000	5.802374000
H	0.000000000	-4.509759000	-5.802374000
H	2.142119000	2.739124000	2.503710000
H	2.142119000	2.739124000	-2.503710000
H	2.142119000	-2.739124000	2.503710000
H	2.142119000	-2.739124000	-2.503710000
H	2.152195000	3.922908000	4.698826000
H	2.152195000	3.922908000	-4.698826000
H	2.152195000	-3.922908000	4.698826000
H	2.152195000	-3.922908000	-4.698826000
H	-2.142119000	2.739124000	2.503710000
H	-2.142119000	2.739124000	-2.503710000
H	-2.142119000	-2.739124000	2.503710000
H	-2.142119000	-2.739124000	-2.503710000
H	-2.152195000	3.922908000	4.698826000
H	-2.152195000	3.922908000	-4.698826000
H	-2.152195000	-3.922908000	4.698826000
H	-2.152195000	-3.922908000	-4.698826000
N	0.000000000	1.975672000	1.135584000
N	0.000000000	1.975672000	-1.135584000
N	0.000000000	-1.975672000	1.135584000
N	0.000000000	-1.975672000	-1.135584000

5. $Mo_2[HC(NPh)_2]_2$, D_{2h}, q = 0, S = 0

Mo	0.000000000	0.000000000	0.998390000
Mo	0.000000000	0.000000000	-0.998390000
C	0.000000000	2.801415000	0.000000000
C	0.000000000	2.848420000	2.366991000
C	0.000000000	2.848420000	-2.366991000
C	0.000000000	4.184252000	4.832251000
C	0.000000000	4.184252000	-4.832251000
C	0.000000000	-2.801415000	0.000000000
C	0.000000000	-2.848420000	2.366991000
C	0.000000000	-2.848420000	-2.366991000
C	0.000000000	-4.184252000	4.832251000
C	0.000000000	-4.184252000	-4.832251000
C	1.207459000	3.851780000	4.213513000
C	1.207459000	3.851780000	-4.213513000

C	1.207459000	-3.851780000	4.213513000
C	1.207459000	-3.851780000	-4.213513000
C	1.209929000	3.186285000	2.987054000
C	1.209929000	3.186285000	-2.987054000
C	1.209929000	-3.186285000	2.987054000
C	1.209929000	-3.186285000	-2.987054000
C	-1.207459000	3.851780000	4.213513000
C	-1.207459000	3.851780000	-4.213513000
C	-1.207459000	-3.851780000	4.213513000
C	-1.207459000	-3.851780000	-4.213513000
C	-1.209929000	3.186285000	2.987054000
C	-1.209929000	3.186285000	-2.987054000
C	-1.209929000	-3.186285000	2.987054000
C	-1.209929000	-3.186285000	-2.987054000
H	0.000000000	3.894611000	0.000000000
H	0.000000000	4.699051000	5.790020000
H	0.000000000	4.699051000	-5.790020000
H	0.000000000	-3.894611000	0.000000000
H	0.000000000	-4.699051000	5.790020000
H	0.000000000	-4.699051000	-5.790020000
H	2.142061000	2.912693000	2.499757000
H	2.142061000	2.912693000	-2.499757000
H	2.142061000	-2.912693000	2.499757000
H	2.142061000	-2.912693000	-2.499757000
H	2.151954000	4.108945000	4.687958000
H	2.151954000	4.108945000	-4.687958000
H	2.151954000	-4.108945000	4.687958000
H	2.151954000	-4.108945000	-4.687958000
H	-2.142061000	2.912693000	2.499757000
H	-2.142061000	2.912693000	-2.499757000
H	-2.142061000	-2.912693000	2.499757000
H	-2.142061000	-2.912693000	-2.499757000
H	-2.151954000	4.108945000	4.687958000
H	-2.151954000	4.108945000	-4.687958000
H	-2.151954000	-4.108945000	4.687958000
H	-2.151954000	-4.108945000	-4.687958000
N	0.000000000	2.119136000	1.145265000
N	0.000000000	2.119136000	-1.145265000
N	0.000000000	-2.119136000	1.145265000
N	0.000000000	-2.119136000	-1.145265000

6. $W_2[HC(NPh)_2]_2$, D_{2h}, q = 0, S = 0

W	0.000000000	0.000000000	1.050383000
W	0.000000000	0.000000000	-1.050383000
C	0.000000000	2.785234000	0.000000000
C	0.000000000	2.814685000	2.374508000
C	0.000000000	2.814685000	-2.374508000
C	0.000000000	4.140398000	4.846256000
C	0.000000000	4.140398000	-4.846256000
C	0.000000000	-2.785234000	0.000000000

C	0.000000000	-2.814685000	2.374508000
C	0.000000000	-2.814685000	-2.374508000
C	0.000000000	-4.140398000	4.846256000
C	0.000000000	-4.140398000	-4.846256000
C	1.209408000	3.807641000	4.227375000
C	1.209408000	3.807641000	-4.227375000
C	1.209408000	-3.807641000	4.227375000
C	1.209408000	-3.807641000	-4.227375000
C	1.211327000	3.145528000	2.996962000
C	1.211327000	3.145528000	-2.996962000
C	1.211327000	-3.145528000	2.996962000
C	1.211327000	-3.145528000	-2.996962000
C	-1.209408000	3.807641000	4.227375000
C	-1.209408000	3.807641000	-4.227375000
C	-1.209408000	-3.807641000	4.227375000
C	-1.209408000	-3.807641000	-4.227375000
C	-1.211327000	3.145528000	2.996962000
C	-1.211327000	3.145528000	-2.996962000
C	-1.211327000	-3.145528000	2.996962000
C	-1.211327000	-3.145528000	-2.996962000
H	0.000000000	3.875575000	0.000000000
H	0.000000000	4.653554000	5.805144000
H	0.000000000	4.653554000	-5.805144000
H	0.000000000	-3.875575000	0.000000000
H	0.000000000	-4.653554000	5.805144000
H	0.000000000	-4.653554000	-5.805144000
H	2.141776000	2.873029000	2.506086000
H	2.141776000	2.873029000	-2.506086000
H	2.141776000	-2.873029000	2.506086000
H	2.141776000	-2.873029000	-2.506086000
H	2.153558000	4.062173000	4.704056000
H	2.153558000	4.062173000	-4.704056000
H	2.153558000	-4.062173000	4.704056000
H	2.153558000	-4.062173000	-4.704056000
H	-2.141776000	2.873029000	2.506086000
H	-2.141776000	2.873029000	-2.506086000
H	-2.141776000	-2.873029000	2.506086000
H	-2.141776000	-2.873029000	-2.506086000
H	-2.153558000	4.062173000	4.704056000
H	-2.153558000	4.062173000	-4.704056000
H	-2.153558000	-4.062173000	4.704056000
H	-2.153558000	-4.062173000	-4.704056000
N	0.000000000	2.089466000	1.147122000
N	0.000000000	2.089466000	-1.147122000
N	0.000000000	-2.089466000	1.147122000
N	0.000000000	-2.089466000	-1.147122000

7. $Cr_2[HC(NH)_2]_2$, D_{2h}, q = 0, *S* = 0

Cr	0.000000000	0.000000000	0.827857000
Cr	0.000000000	0.000000000	-0.827857000

C	2.677154000	0.000000000	0.000000000
C	-2.677154000	0.000000000	0.000000000
H	2.545306000	0.000000000	1.971013000
H	2.545306000	0.000000000	-1.971013000
H	3.773890000	0.000000000	0.000000000
H	-2.545306000	0.000000000	1.971013000
H	-2.545306000	0.000000000	-1.971013000
H	-3.773890000	0.000000000	0.000000000
N	1.977108000	0.000000000	1.131771000
N	1.977108000	0.000000000	-1.131771000
N	-1.977108000	0.000000000	1.131771000
N	-1.977108000	0.000000000	-1.131771000

8. $Mo_2[HC(NH)_2]_2$, D_{2h}, q = 0, *S* = 0

Mo	0.000000000	0.000000000	0.999187000
Mo	0.000000000	0.000000000	-0.999187000
C	2.804875000	0.000000000	0.000000000
C	-2.804875000	0.000000000	0.000000000
H	2.711634000	0.000000000	1.963420000
H	2.711634000	0.000000000	-1.963420000
H	3.900619000	0.000000000	0.000000000
H	-2.711634000	0.000000000	1.963420000
H	-2.711634000	0.000000000	-1.963420000
H	-3.900619000	0.000000000	0.000000000
N	2.116332000	0.000000000	1.141483000
N	2.116332000	0.000000000	-1.141483000
N	-2.116332000	0.000000000	1.141483000
N	-2.116332000	0.000000000	-1.141483000

9. $W_2[HC(NH)_2]_2$, D_{2h}, q = 0, *S* = 0

W	0.000000000	0.000000000	1.049119000
W	0.000000000	0.000000000	-1.049119000
C	2.784857000	0.000000000	0.000000000
C	-2.784857000	0.000000000	0.000000000
H	2.683141000	0.000000000	1.967380000
H	2.683141000	0.000000000	-1.967380000
H	3.877875000	0.000000000	0.000000000
H	-2.683141000	0.000000000	1.967380000
H	-2.683141000	0.000000000	-1.967380000
H	-3.877875000	0.000000000	0.000000000
N	2.086788000	0.000000000	1.144608000
N	2.086788000	0.000000000	-1.144608000
N	-2.086788000	0.000000000	1.144608000
N	-2.086788000	0.000000000	-1.144608000

10. Example input for molecule with q = 0, *S* = 0

```
Title Quint

COMMENT
no comment
END

UNITS
  length angstrom
END

Atoms Cartesian
*** add xyz coordinates
end

SYMMETRY D(2h)

CHARGE 0

INTEGRATION
  accint 6.0
End

RELATIVISTIC Scalar ZORA

GEOMETRY
  Iterations 500
  Converge e=1.0E-3 grad=1.0E-3 rad=1.0E-2
End

SCF
Iterations 500
Converge 1.0E-7
END

BASIS
  Type ZORA/TZ2P
  Core none
END

XC
  gga OLYP
  DISPERSION Grimme3
END

endinput
```
